# The potential mechanism of Longsheyangquan Decoction on the treatment of bladder cancer: Systemic network pharmacology and molecular docking

**DOI:** 10.3389/fphar.2022.932039

**Published:** 2022-07-14

**Authors:** Zhang Cheng, Fangdie Ye, Chenyang Xu, Yingchun Liang, Zheyu Zhang, Xinan Chen, Xiyu Dai, Yuxi Ou, Zezhong Mou, Weijian Li, Yiling Chen, Quan Zhou, Lujia Zou, Shanhua Mao, Haowen Jiang

**Affiliations:** ^1^ Department of Urology, Huashan Hospital, Fudan University, Shanghai, China; ^2^ Fudan Institute of Urology, Huashan Hospital, Fudan University, Shanghai, China; ^3^ National Clinical Research Center for Aging and Medicine, Huashan Hospital, Fudan University, Shanghai, China

**Keywords:** bladder cancer, Longsheyangquan Decoction, target, network pharmacology, molecular docking, traditional Chinese medicine

## Abstract

Our goal was to explore the bioactive constituents of Longsheyangquan (LSYQ) Decoction and elucidate its mechanisms on the treatment of bladder cancer (BCa). A total of 38 compounds were selected based on their pharmacokinetic properties in three large traditional Chinese medicine (TCM) databases. 654 putative targets of LSYQ Decoction were predicted using a structure-based, reverse-docking algorithm online, of which 343 overlapped with BCa-related protein-coding genes. The protein-protein interaction (PPI) network was constructed to perform module analysis for further Gene Ontology (GO) annotations and Kyoto Encyclopedia Genes and Genomes (KEGG) pathway enrichment analysis, which identified *CDK2, EGFR, MMP9* and *PTGS2* as hub targets. The TCM-compound-target network and compound-target-pathway network together revealed that quercetin, diosmetin, enhydrin and luteolin were the main components of LSYQ Decoction. Finally, molecular docking showed the affinity between the key compounds and the hub target proteins to verify the accuracy of drug target prediction in the first place. The present study deciphered the core components and targets of LSYQ Decoction on the treatment of BCa in a comprehensive systemic pharmacological manner.

## Introduction

It is estimated that there were 573,278 new cases and 212,536 disease-related deaths of bladder cancer worldwide in 2020 alone, making it the 10th most commonly diagnosed cancer globally and the most common malignancy of the urinary system (Sung et al., 2021). The past 2 decades have witnessed a transition of the cancer spectrum in China that is shifting from a less-developed country to a developed one (Feng et al., 2019). At the same time, the incidence of BCa has increased to a level similar to that of Western Europe and the United States, due to an increasingly westernized lifestyle and an upward trend of smoking among women (Feng et al., 2019). Despite being confined to the mucosa (stage Ta, carcinoma *in situ*) or submucosa (stage T1) at the early stage, BCa often recurs and requires frequent imaging examinations, such as urinary ultrasonography, contrast-enhanced CT, or sometimes even transurethral cystoscopy. However, approximately 30% of non-muscle invasive bladder cancers would gradually progress into muscle-invasive ones during the course of the disease (van der Heijden and Witjes, 2009). Postoperative immediate and regular intravesical administration of Bacillus Calmette-Guerin, pirarubicin, mitomycin or gemcitabine could effectively kill the residual tumor cells after transurethral resection of bladder tumors for superficial BCa (Babjuk et al., 2022). Radical cystectomy or bladder-sparing surgery followed by platinum-based chemotherapy remains the first-line treatment for advanced-stage patients. Although neoadjuvant chemotherapy and especially immunotherapy with checkpoint inhibitors have improved the prognosis of muscle-invasive and metastatic BCa in recent years (Singh and Black, 2016; Burdett et al., 2022), the 5-year overall survival and response rate to the immunotherapy remain low (Gakis, 2020). In addition to the uncertain therapeutic outcome, the local and systemic side effects associated with chemo- or immunotherapy have become a major reason for discontinued treatment and have limited their further clinical applications (Singh and Black, 2016). Therefore, it is of critical urgency to develop novel cures with high efficacy while reducing toxicity.

Traditional Chinese medicine usually consists of various herbal medicines derived from natural plants, and has been used for thousands of years to treat different types of diseases, with the goal of establishing the dynamic balance of the whole body. Longsheyangquan (LSYQ) Decoction whose composition is primarily *Solanum Nigrum Linn., Duchesnea Indica Focke, Herba Solani Lyrati, Spora Lygodii, Smilacis Glabrae Rhizoma* and *Juncus effusus L.*, is an empirical prescription for preventing postoperative BCa recurrence on the basis of TCM theory (Zhou, 2006). While some of these ingredients have been proven to exert anti-cancer effect, the underlying mechanisms of LSYQ Decoction as a whole and its promising therapeutic effect on BCa are still largely unclear.

Network pharmacology, first proposed by Dr. Hopkins, is an efficient tool for systemic pharmacological research in which the interactions among target genes, pathways, diseases, and drugs are studied in detail using a network-based method to unravel the mechanisms of complex preparations at multiple levels (Hopkins, 2007). Due to the in-depth analysis and holistic view shared with classical Chinese medicine, it is quite suitable for the research of TCM herbs and formulas. Molecular docking is a frequently-used method in the field of structure-based drug design that predicts the preferred conformation when a small molecule ligand binds to a target to form a stable complex, and the strength of binding, i.e., the affinity between them, can be calculated (Lengauer and Rarey, 1996).

Herein, we aimed to comprehensively investigate LSYQ Decoction as well as its influence on BCa using an *in-silico* approach, as neither had been reported before. Initially, we screened out and selected the putative bioactive components of LSYQ Decoction. Then, we acquired the overlap between the drug targets and BCa-related target genes for further GO and KEGG enrichment analyses. Meanwhile, we visualized the TCM-compound-target network as well as the compound-target-pathway network to provide a general overview of the potential anti-tumor properties and molecular mechanisms of LSYQ Decoction. Finally, we employed molecular docking to confirm the affinity between the core components and the hub targets. The current study not only elucidated the possible mechanisms of LSYQ Decoction on the treatment of BCa, but also serve to promote more research into TCM in order to discover highly effective herbal medications with mild side effects and relatively low research and development investment.

**FIGURE 1:**
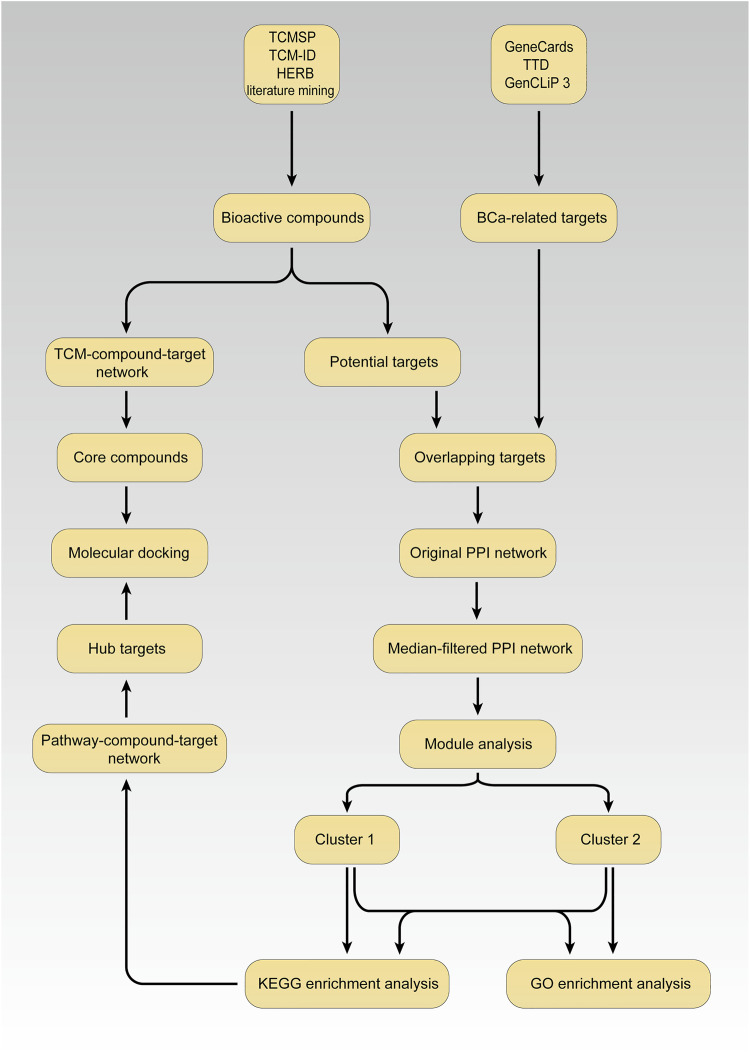
The scheme for investigation of mechanisms of LSYQ Decoction on the treatment of BCa.

A systemic scheme of the pharmacological network for the present study is illustrated in Figure 1.

## Methods

### Chemical compounds of LSYQ Decoction and targets prediction

The Traditional Chinese Medicine System Pharmacology (TCMSP) online database (https://tcmsp-e.com/tcmsp.php) was utilized in search of chemical constituents of LSYQ Decoction, which is composed of *Solanum Nigrum Linn.*, *Duchesnea Indica Focke*, *Herba Solani Lyrati*, *Spora Lygodii*, *Smilacis Glabrae Rhizoma* and *Juncus effusus L*. The database integrates TCMs, chemical components and their pharmacokinetic properties, including oral bioavailability (OB), gastrointestinal absorption, blood-brain barrier permeability, drug-likeness (DL) etc. The active compounds were selected by criteria as follows: OB ≥ 30%, DL ≥ 0.18, molecular weight ≤500 Dal, hydrogen bond donors ≤5, hydrogen bond acceptors ≤10 and AlogP ≤5, with the last four corresponding to the “Rule of Five” first raised by Lipinski in 1997 (Lipinski, 2016). We then searched HERB and the Traditional Chinese Medicine Information Database (TCM-ID) for additional chemical constituents as supplementary, the former launched by the Institute of Computing Technology of Chinese Academy of Sciences as well as Beijing University of Chinese Medicine, and the latter by the National University of Singapore. Literature mining was also conducted on the China National Knowledge Internet (CNKI, https://www.cnki.net/) for more possible constituents, especially in the case of *Duchesnea Indica Focke* whose components were re-input into the TCMSP to obtain pharmacokinetic properties for next-step selection. Additional compounds were input into SwissADME (http://www.swissadme.ch/) (Daina et al., 2017)for pharmacokinetic and drug-likeness parameters. Only compounds with high gastrointestinal absorption that met at least three of the five drug-likeness principles were selected.

Targets for a total of 38 potentially-bioactive compounds were predicted by SwissTargetPrediction (http://www.swisstargetprediction.ch/) (Daina et al., 2019), which determines the most likely protein targets of a small molecule based on a combination of 2D and 3D similarity. *Homo Sapiens* was chosen as the only species.

### Construction of TCM-compound-target network

Cytoscape software (version 3.8.0) was used to vividly display the relationship between TCMs, potentially-bioactive compounds and targets associated with the compounds.

### Identification of BCa-related targets

Human BCa-related targets were searched using the following terms: “bladder cancer” AND “bladder carcinoma” AND “malignant neoplasm of bladder” AND “bladder carcinoma *in situ*”. Targets were retrieved from GeneCards (https://www.genecards.org/), Therapeutic Target Database (TTD, http://db.idrblab.net/ttd/) and GenCLiP 3 (http://ci.smu.edu.cn/genclip3/analysis.php). For GeneCards, only targets with relevance scores greater than the median were selected. The universal Protein Resources (UniProt, https://www.uniprot.org/) was subsequently used to distinguish protein-coding target genes from non-coding ones.

### Establishment of protein-protein interaction network and module analysis

Overlapping targets of LSYQ Decoction and BCa were acquired and considered as candidate targets by means of a Venn diagram via an online plotting resource (http://jvenn.toulouse.inra.fr/app/index.html). An original PPI network involving candidate targets was created by the Search Tool for the Retrieval of Interacting Genes (STRING, https://string-db.org/) in order to explore the relationship between the imported protein-coding genes. Afterwards, the original PPI network was analyzed by Cytoscape and its CytoNCA plugin. Nodes whose Betweenness, Closeness and Degree were less than the corresponding medians were filtered out. Furthermore, the Molecular Complex Detection (MCODE) plugin helped us to select and visualize significant modules with criteria as follows: degree cut off = 2, node score cut off = 0.2, K-core = 2 and maximum depth = 100. Modules with score ≥10 were included for further analyses.

### Functional annotation and enrichment analyses

Gene ontology analysis is a widely-accepted approach to define and describe genes across species from three aspects, namely molecular function (MF), biological process (BP), and cellular component (CC). KEGG is in fact an enormous collection of databases involving genomes, drugs, enzymes, biological pathways etc. Herein, the Metascape (https://metascape.org/gp/index.html#/main/step1) was applied to perform GO and KEGG enrichment analyses for two clusters with adjusted *p* values <0.01 as the cut-off.

### Construction of compound-target-pathway network

A network was plotted by Cytoscape for the top 25 KEGG pathways in the top-ranked module with the corresponding targets and chemical compounds so as to highlight the hub target genes and the core compounds.

### Molecular docking analysis

Molecular docking could intuitively display the interaction between the compounds and their potential target proteins with the help of Autodock (version 4.2.6). The 3D structures of the core compounds were downloaded from PubChem (https://pubchem.ncbi.nlm.nih.gov/), while the crystal structures of human proteins encoded by the hub genes were obtained from the RCSB Protein Data Bank (PDB, https://www.rcsb.org/). The protein structures were then pre-processed by removing the irrelevant water molecules and ligands, adding hydrogen atoms, and merging non-polar hydrogen atoms. After adding hydrogen atoms for the compounds, protein-compound docking was conducted with the method of Lamarckian genetic algorithm in Autodock Tools (version 1.5.6). The number of runs for each docking was set to 50 as officially recommended, in order to gain the best affinity (i.e., the lowest binding energy), the conformation of which was then visualized in PyMOL software (version 2.2.0).

## Results

### Chemical compounds and potential targets of LSYQ Decoction

38 of the 386 compounds in LSYQ Decoction were identified as drug-like molecules (Tables 1, 2) and had their putative targets successfully predicted by SwissTargetPrediction, which yielded 654 human proteins after the removal of duplicates. There were six components in *Solanum Nigrum Linn.*, four components in *Duchesnea Indica Focke*, three components in *Herba Solani Lyrati*, four components in *Spora Lygodi*, five components in *Smilacis Glabrae Rhizoma* and 19 components in *Juncus effusus L*. It is worth noting that quercetin and diosgenin were present in two or three herbal medicines.

**TABLE 1:** Bioactive compounds retrieved from TCMSP.

No.	Name	MF	MW	OB (%)	DL
*Solanum Nigrum Linn*					
Mol-1	Quercetin	C_15_H_10_O_7_	302.25	46.43	0.28
Mol-2	Diosgenin	C_27_H_42_O_3_	414.69	80.88	0.81
Mol-3	Medioresinol	C_21_H_24_O_7_	388.45	57.2	0.62
Mol-4	Solanocapsine	C_27_H_46_N_2_O_2_	430.75	52.94	0.67
*Duchesnea Indica Focke*					
Mol-7	Farrerol	C_17_H_16_O_5_	300.33	42.65	0.26
*Herba Solani Lyrati*					
Mol-1	Quercetin	C_15_H_10_O_7_	302.25	46.43	0.28
Mol-11	Sophocarpine	C_15_H_22_N_2_O	246.39	64.26	0.25
*Spora Lygodii*					
Mol-13	Kaempferol	C_15_H_10_O_6_	286.25	41.88	0.24
Mol-14	Acacetin	C_16_H_12_O_5_	284.28	34.97	0.24
Mol-15	Diosmetin	C_16_H_12_O_6_	300.28	31.14	0.27
*Smilacis Glabrae Rhizoma*					
Mol-1	Quercetin	C_15_H_10_O_7_	302.25	46.43	0.28
Mol-2	Diosgenin	C_27_H_42_O_3_	414.69	80.88	0.81
Mol-17	Naringenin	C_15_H_12_O_5_	272.27	59.29	0.21
Mol-18	Enhydrin	C_23_H_28_O_10_	464.51	40.56	0.74
*Juncus effusus L*					
Mol-20	Luteolin	C_15_H_10_O_6_	286.25	36.16	0.25
Mol-21	4-Ethenyl-7-hydroxy-8-methyl-9,10-dihydrophenanthrene-1-carboxylic acid	C_18_H_16_O_3_	280.36	56.99	0.27
Mol-22	2,8-Dihydroxy-1,7-dimethyl-6-ethenyl-10,11-dihydrodibenz [b,f]-oxepin	C_18_H_18_O_3_	282.36	58.95	0.27
Mol-23	1,8-Dimethyl-4-vinyl-9,10-dihydrophenanthrene-2,7-diol	C_18_H_18_O_2_	266.36	42.1	0.23
Mol-24	2,8-Dihydroxy-1,6-dimethyl-5-ethenyl-9,10-dihydrophenanthrene	C_18_H_18_O_2_	266.36	35.77	0.23
Mol-25	Dehydroeffusal	C_16_H_12_O_3_	252.28	79.55	0.21
Mol-26	5-(1-Methoxyethyl)-2,6-dihydroxy-1,7-dimethyl-9,10-dihydrophenanthrene	C_19_H_22_O_3_	298.41	35.77	0.29
Mol-27	3,7-Dihydroxy-2,8-dimethyl-9,10-dihydrophenanthrene-4-carbaldehyde	C_17_H_16_O_3_	268.33	40.49	0.23
Mol-28	7-Hydroxy-2-methoxy-1,8-dimethyl-9,10-dihydrophenanthrene-4-carbaldehyde	C_18_H_18_O_3_	282.36	60.42	0.26
Mol-29	Effusol	C_17_H_16_O_2_	252.33	56.43	0.2
Mol-30	1-(3,7-Dihydroxy-2,8-dimethyl-9,10-dihydrophenanthren-4-yl)ethanone	C_18_H_18_O_3_	282.36	44.72	0.26
Mol-31	5-(1-Hydroxyethyl)-2,6-dihydroxy-1,7-dimethyl-9,10-dihydrophenanthrene	C_18_H_20_O_3_	284.38	42.31	0.26
Mol-32	Eriodictyol	C_15_H_12_O_6_	288.27	71.79	0.24

MF, molecular formula; MW, molecular weight; OB, oral bioavailability; DL, Drug-likeness.

**TABLE 2:** Bioactive compounds retrieved from other two databases and through literature mining.

No.	Name	MF	MW	Drug-likeness				
				Lipinski	Ghose	Veber	Egan	Muegge
*Solanum Nigrum Linn*								
Mol-5	15α-Hydroxysolasodine	C_27_H_43_NO_3_	429.64	Yes	No	Yes	Yes	Yes
Mol-6	N-Methyl-solasodine	C_28_H_45_NO_2_	427.66	Yes	No	Yes	Yes	No
*Duchesnea Indica Focke*								
Mol-8	Iminodiacetic acid	C_4_H_7_NO_4_	113.10	Yes	No	Yes	Yes	No
Mol-9	Betuloside	C_16_H_24_O_7_	328.36	Yes	No	Yes	Yes	Yes
Mol-10	Methyl brevifolincarboxylate	C_14_H_10_O_8_	306.22	Yes	Yes	No	Yes	Yes
*Herba Solani Lyrati*								
Mol-12	15α-Hydroxytomatidine	C_27_H_45_NO_3_	431.73	Yes	No	Yes	Yes	No
*Spora Lygodii*								
Mol-16	2-Anilino-1,4-naphthoquinone	C_16_H_11_NO_2_	249.26	Yes	Yes	Yes	Yes	Yes
*Smilacis Glabrae Rhizoma*								
Mol-19	7,6′-Dihydroxy-3′-methoxyisoflavone	C_16_H_12_O_5_	284.28	Yes	Yes	Yes	Yes	Yes
*Juncus effusus L*								
Mol-33	2,6-Dihydroxy-1,7-dimethyl-5-ethenyl-9,10-dihydrophenanthrene	C_18_H_18_O_2_	266.36	Yes	Yes	Yes	Yes	Yes
Mol-34	1-Methyleffusol	C_18_H_18_O_2_	266.36	Yes	Yes	Yes	Yes	Yes
Mol-35	2,6-Dihydroxy-1,7-dimethyl-9,10-dihydrophenanthrene	C_16_H_16_O_2_	240.30	Yes	Yes	Yes	Yes	Yes
Mol-36	2′,5′,5,7-Tetrahydroxyflavone	C_15_H_12_O_6_	288.27	Yes	Yes	Yes	Yes	Yes
Mol-37	2,7-Dihydroxy-1,6-dimethyl-pyrene	C_18_H_14_O_2_	262.30	Yes	Yes	Yes	Yes	No
Mol-38	Effusenone A	C_23_H_36_O_5_	392.53	Yes	Yes	Yes	Yes	Yes

MF, molecular formula; MW, molecular weight.

### TCM-compound-target network

**FIGURE 2:**
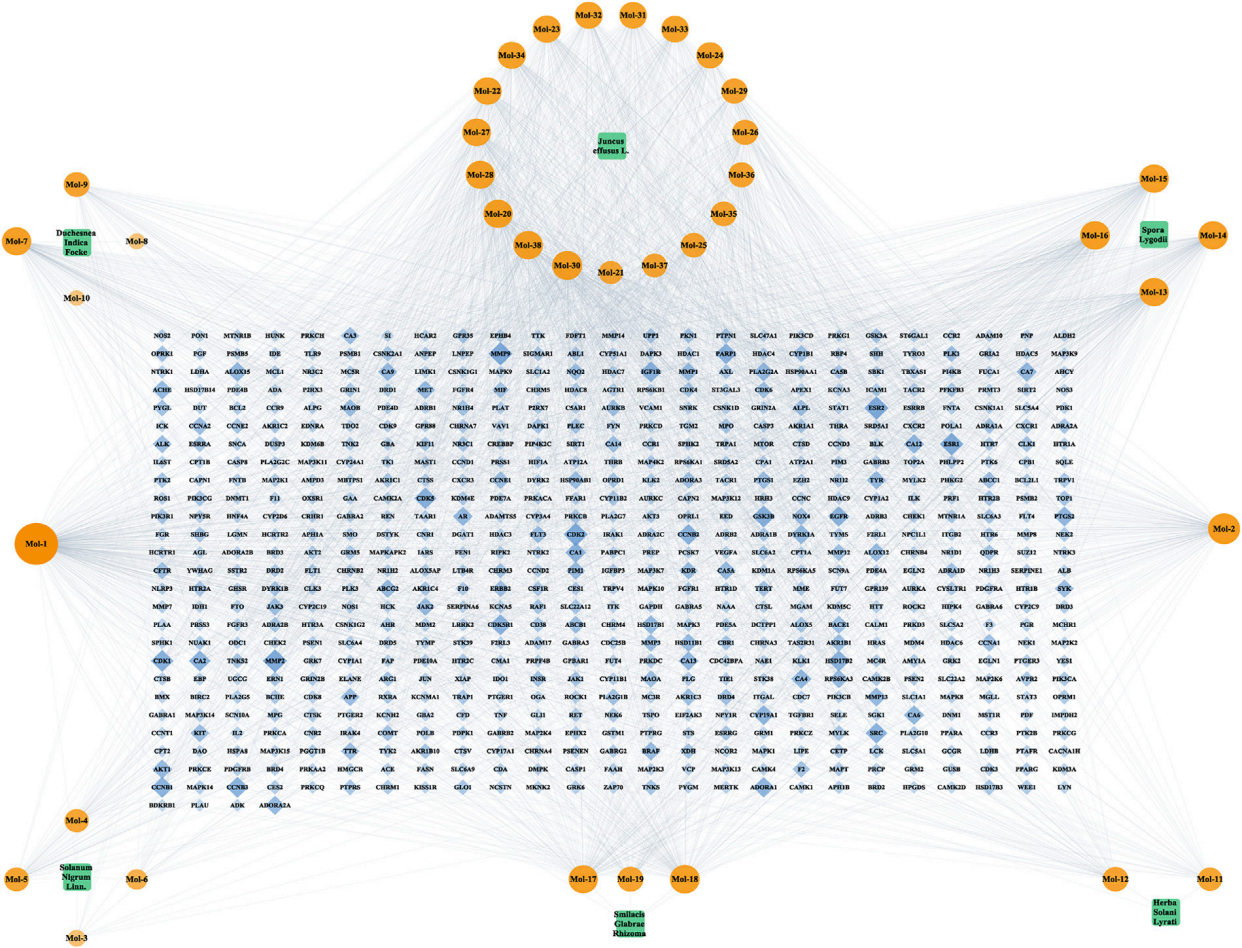
The TCM-compound-target network with 698 nodes and 2,861 edges. TCMs, active compounds and targets were represented by green round rectangular-, orange circular- and blue diamond-shaped nodes, respectively. The greater the Degree value of a node was, the larger the size and lower the transparency were in the network.

The intricate relationship between six TCMs, 38 compounds, and 654 compound-related targets was clearly illustrated by Cytoscape in Figure 2. TCMs, active compounds and targets were represented by green round rectangular-, orange circular- and blue diamond-shaped nodes, respectively. The greater the Degree value of a node was, the larger the size and lower the transparency were in the network. This network emphasized the essence of Chinese medicine formulas, that is, multi-components of a formula exerting multi-target effects.

### Identification of candidate targets

**FIGURE 3:**
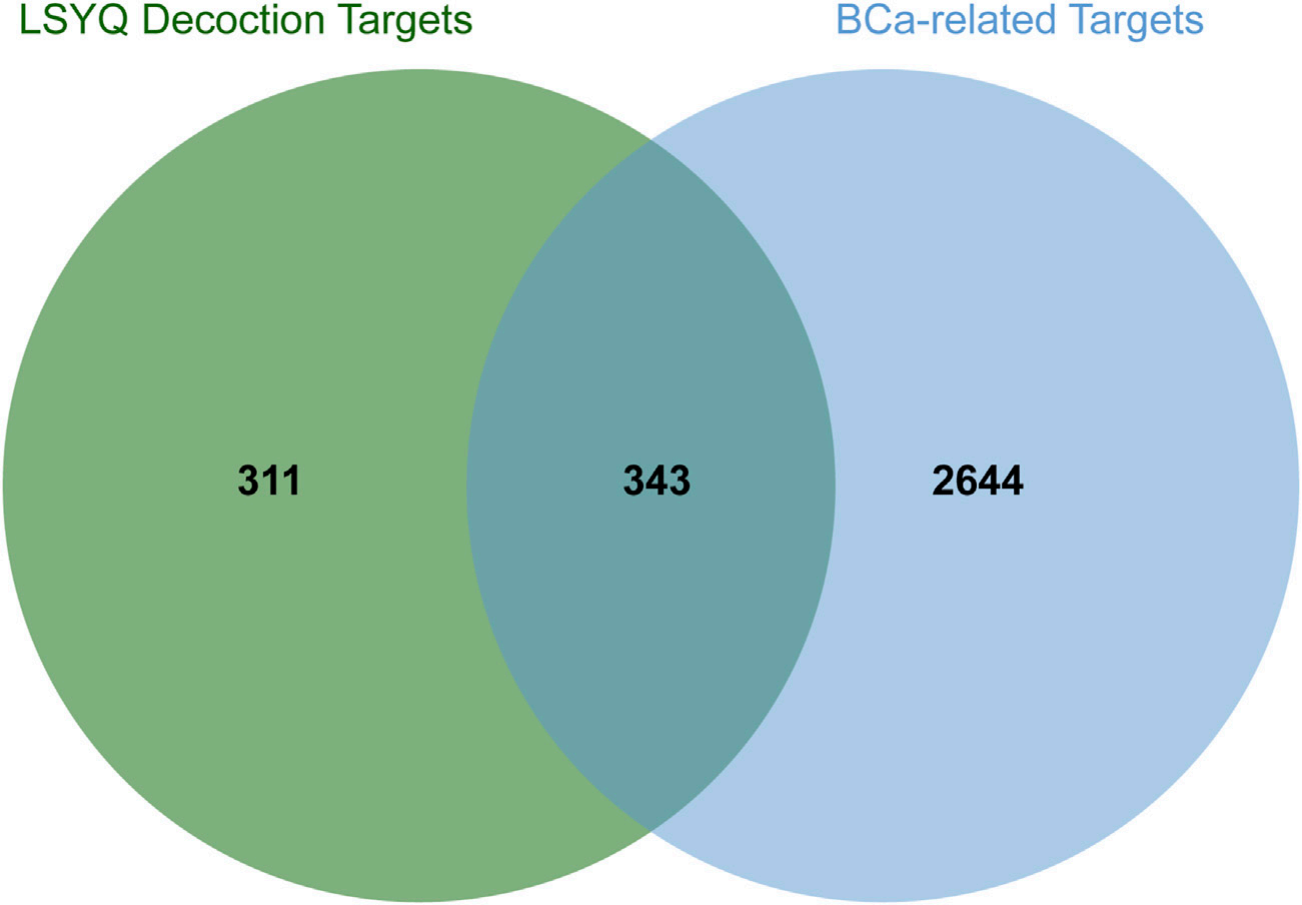
The 343 overlapping targets of LSYQ Decoction and BCa were identified by a Venn diagram.

A total of 7,078 BCa-related genes were extracted from the GeneCards, TTD and GenCLiP three databases. After filtering based on the median of relevance scores and removing duplicates, 2,987 human protein-coding target genes were finally enrolled. Integration of potential TCM target genes and BCa-associated genes presented by a Venn diagram revealed that there were 343 genes in common, indicating their potential therapeutic role in the treatment of BCa by LSYQ Decoction (Figure 3 and Supplementary Table S1).

### Construction and analysis of PPI network

**FIGURE 4:**
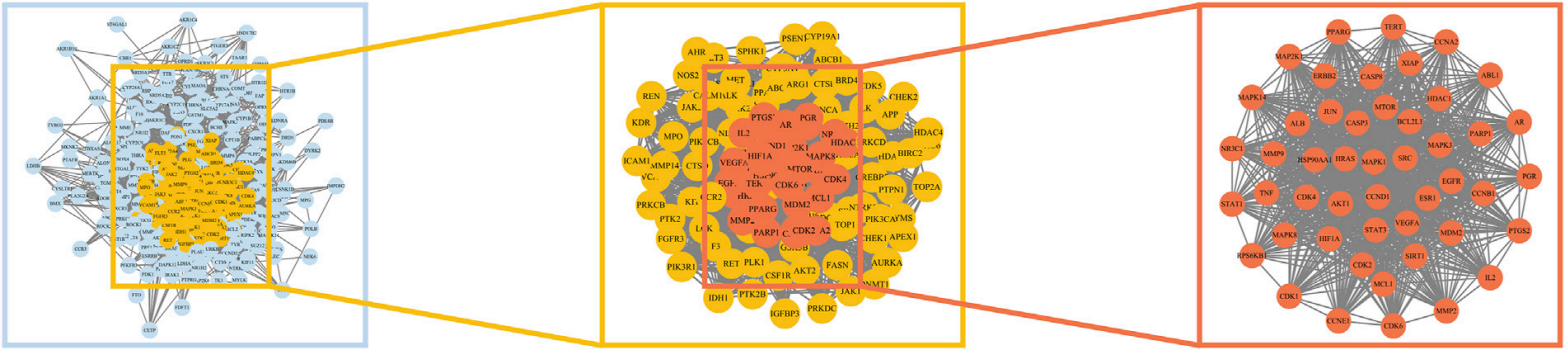
The process of screening the hub targets. The left panel showed the initial PPI network (339 nodes and 6,471 edges) of overlapping candidate targets. The middle panel showed the median-filtered PPI network with 133 nodes and 3,323 edges. The right panel (Cluster One) was generated as a result of MCODE module analysis from the middle panel. Cluster One had 48 nodes and 960 edges with a MCODE score of 40.85.

An original PPI network containing 339 nodes and 6,471 edges was constructed after importing 343 candidate target genes into the STRING website while discarding four disconnected genes. The network owned an average node degree of 37.7 and an average local clustering coefficient of 0.535. The CytoNCA plugin in Cytoscape software was then employed to generate three fundamental topological measures of the centrality of a node in the network: Betweenness, Closeness and Degree. Nodes whose three aforementioned parameters were greater than their corresponding medians formed a core network with 133 nodes and 3,323 edges, in which the MCODE plugin detected two crucial modules with scores ≥10 (Figure 4). The highest scoring module (Cluster One) had a score of 40.85 with 48 nodes and 960 edges, while the other (Cluster Two) had 43 nodes and 261 edges with a score of 12.43 (Figure 6A and Supplementary Table S2).

### Functional annotation and enrichment analyses

**FIGURE 5:**
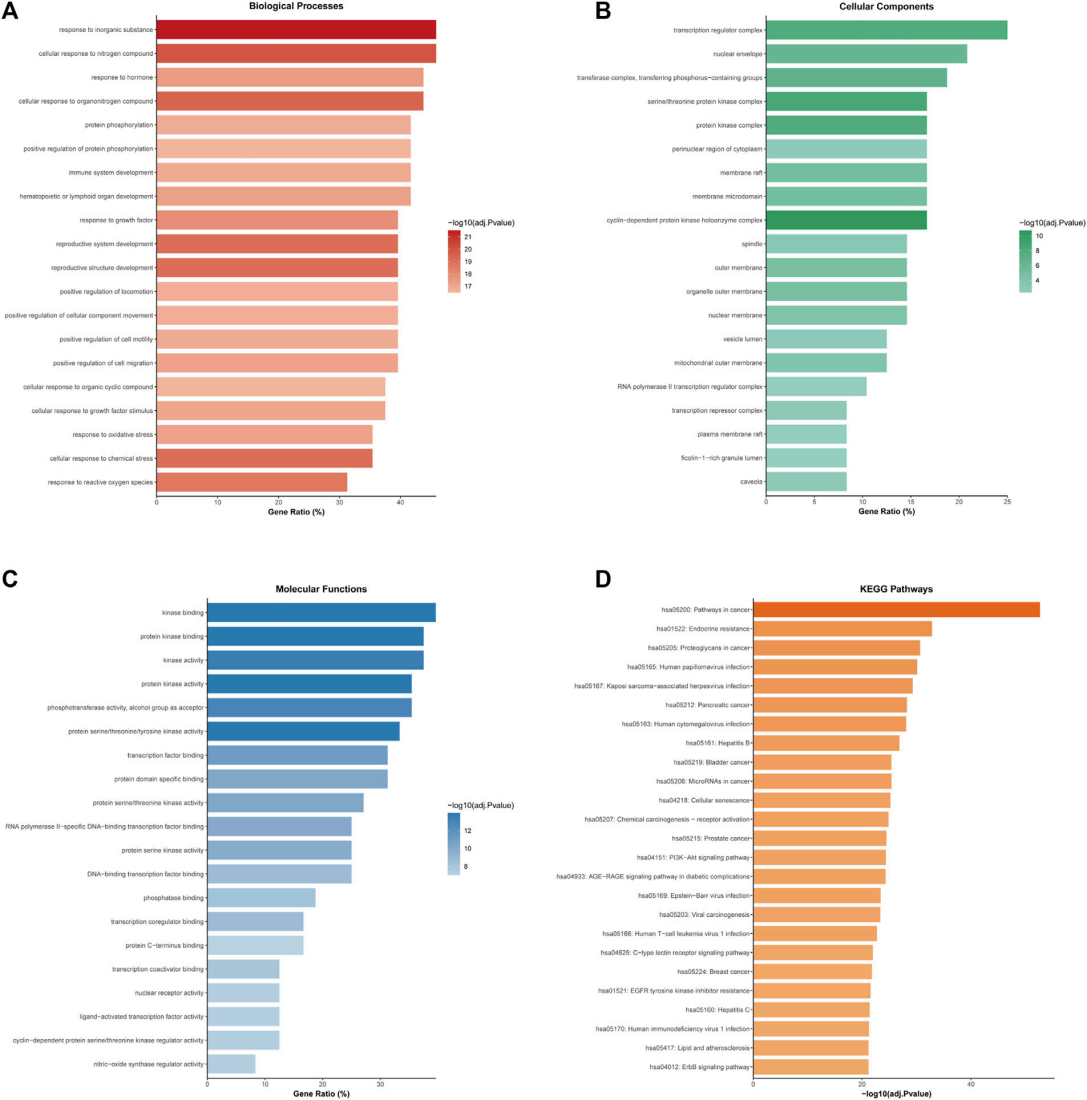
Enrichment analyses of Cluster One for the top 20 GO annotations and top 25 KEGG pathways **(A)** GO analysis of biological process terms **(B)** GO analysis of cellular component terms **(C)** GO analysis of molecular function terms **(D)** KEGG pathway analysis.

**FIGURE 6:**
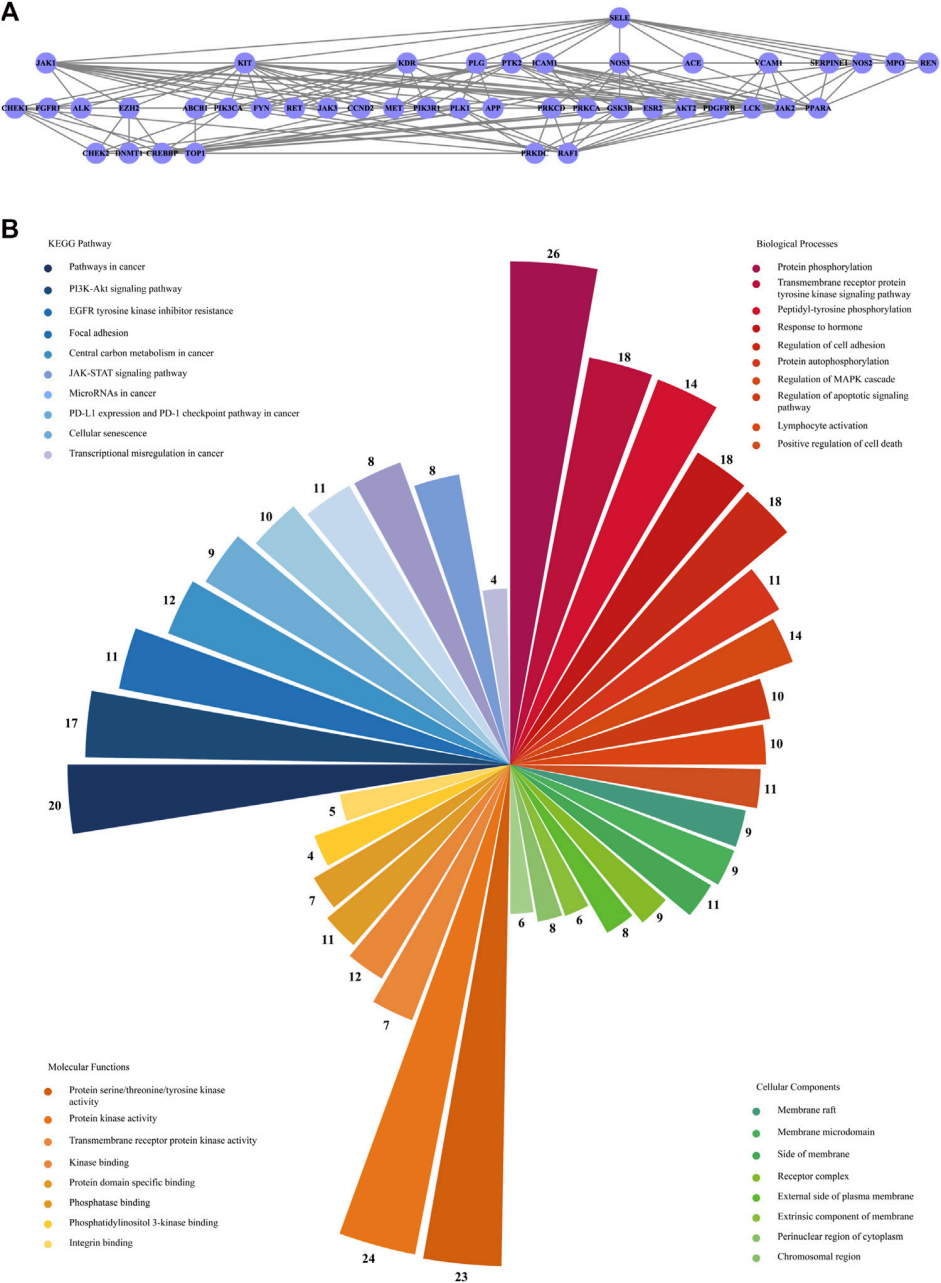
Composition and enrichment analyses of Cluster Two **(A)** Cluster Two had 43 nodes and 261 edges with a MCODE score of 12.43 **(B)** Significantly enriched GO BP/CC/MF terms and KEGG pathways of Cluster Two were displayed in the Nightingale rose diagram, with the number above each petal representing the gene count enriched in this term. The larger the petal, the lower the adjusted *p*-value (all the adjusted *p*-value were under 0.01).

The target genes in two modules were each analyzed on the Metascape website with the log(adjusted *p*-value) ≤ −2 as the cut-off. For Cluster One, the BP analysis revealed that the hub genes were enriched in cellular response to organonitrogen compound, response to reactive oxygen species, positive regulation of cell migration and so on (Figure 5A). The CC analysis showed that hub genes were mainly related with cyclin-dependent protein kinase holoenzyme complex, transcription regulator complex, protein kinase complex and the like (Figure 5B). The MF analysis found that the hub genes were concentrated in protein kinase binding, protein serine/threonine/tyrosine kinase activity, transcription factor binding etc. (Figure 5C). KEGG pathway enrichment analysis indicated that hub genes were remarkably enriched in bladder cancer, endocrine resistance, proteoglycans in cancer and pathways in cancers, such as PI3K-Akt signaling pathway, ErbB signaling pathway, VEGF signaling pathway and MAPK signaling pathway (Figure 5D and Supplementary Table S3). As for Cluster Two, the genes were significantly enriched in pathways in cancer, PI3K-Akt signaling pathway, EGFR tyrosine kinase inhibitor resistance. In addition, a total of 457, eight and 25 terms were enriched in BP, CC and MF, respectively (Figure 6B, full data not shown).

**FIGURE 7:**
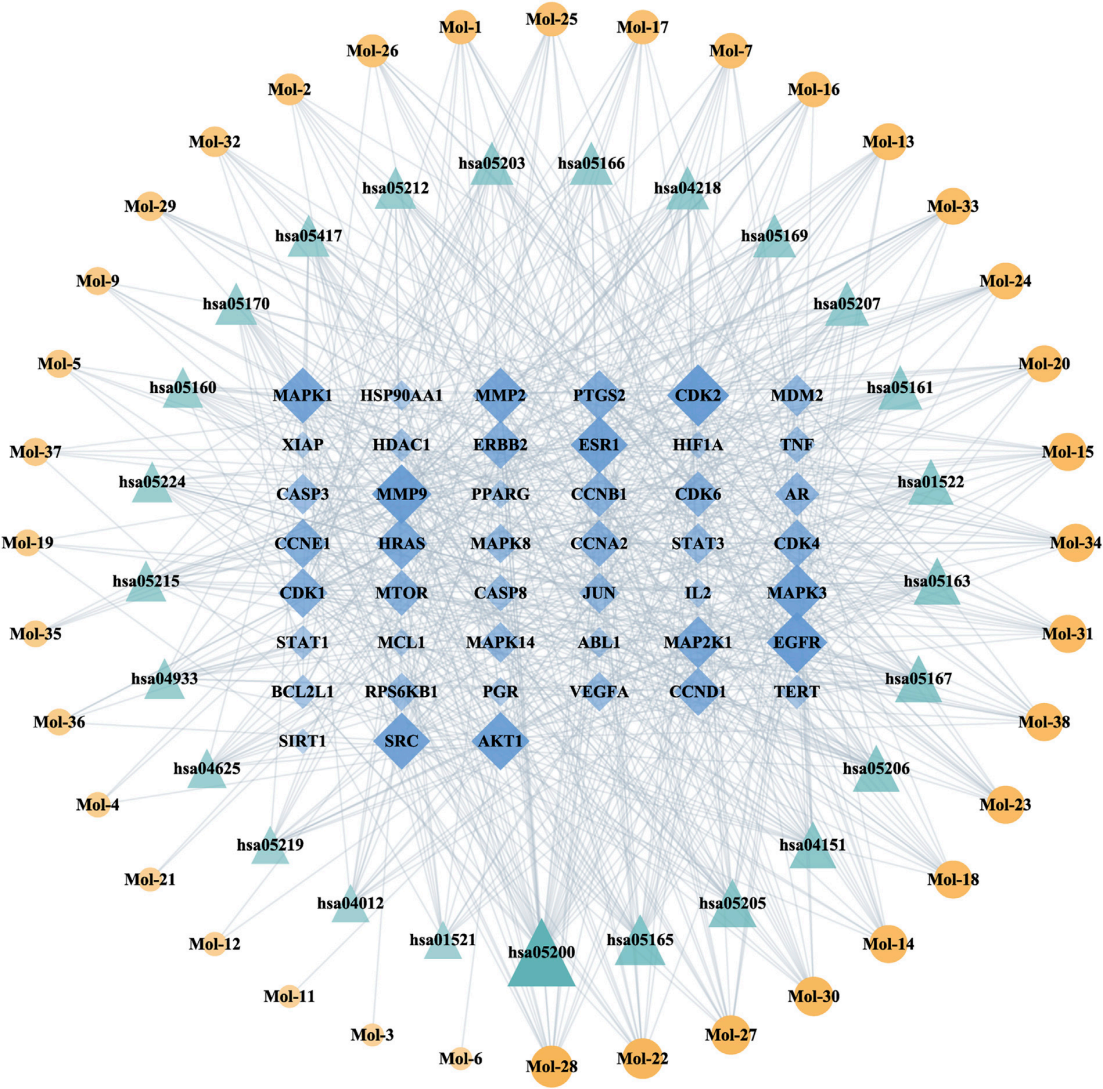
The compound-target-pathway network for the top 25 KEGG pathways in Cluster One. Pathways, compounds and targets were represented by green triangular-, orange circular- and blue diamond-shaped nodes, respectively. The greater the Degree value of a node was, the larger the size and lower the transparency were in the network.

Meanwhile, a network concerning the top 25 KEGG pathways in Cluster One was drawn along with the corresponding targets and drug compounds to accurately illustrate the underlying regulatory mechanisms of LSYQ Decoction for BCa (Figure 7). According to the two PPI networks, the Degree values of Cyclin-dependent kinase 2 (*CDK2*)*,* Epidermal growth factor receptor (*EGFR*)*,* Matrix metalloproteinase 9 (*MMP9*) and Prostaglandin-endoperoxide synthase 2 (*PTGS2*) were among the highest, suggesting their roles as hub targets.

### Molecular docking

**FIGURE 8:**
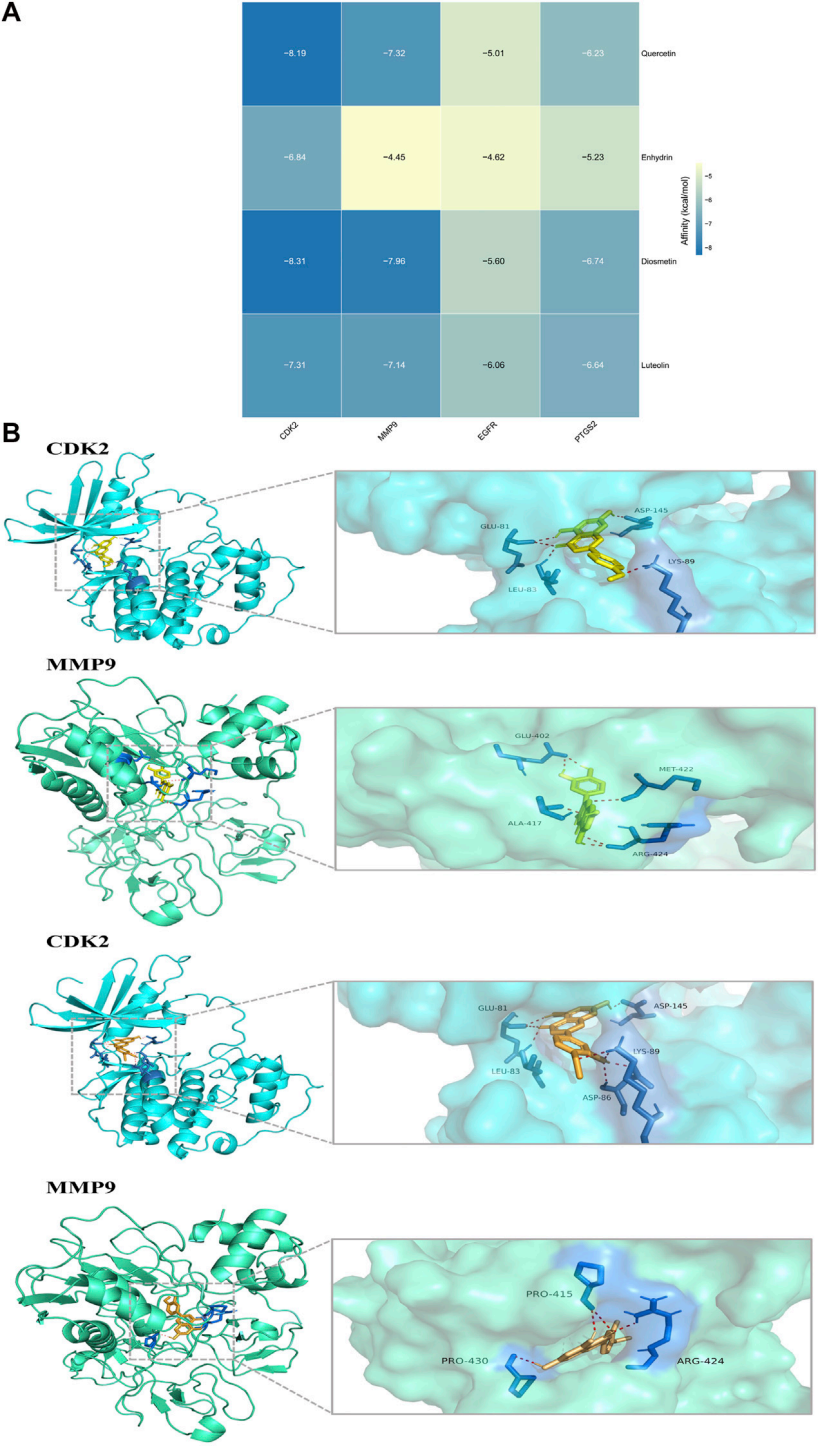
Molecular docking of four typical core compounds with four hub target proteins **(A)** The binding energy heatmap with a deeper color suggesting a more stable binding **(B)** The molecular docking between CDK2, MMP9 and quercetin or diosmetin. The yellow and orange ligands represented quercetin and diosmetin, respectively. The enlarged view on the right showed the interactions of ligands and amino acid residues of CDK2 and MMP9.

To verify the accuracy of SwissTargetPrediction and to ascertain whether key molecules in LSYQ Decoction could directly bind to their protein targets, a widely-accepted molecular docking approach was performed via Autodock. Four typical critical compounds (quercetin, diosmetin, enhydrin and luteolin) attempted to dock with four hub target proteins (CDK2, EGFR, MMP9 and PTGS2). The lower the binding energy, the stronger the bond. The lowest binding energy, in other words, the best affinity, of 16 dockings were shown in a heatmap (Figure 8A). Binding energies lower than −5 kcal/mol, especially < −7 kcal/mol, are considered to form more stable structures than those with higher binding energies. The docking results suggested that all molecules except enhydrin could more or less form stable conformations with four protein receptors, which is consistent with the SwissTargetPrediction results (Figure 8B). Since both quercetin and diosmetin are flavonoids, the sites of the amino acid residues forming hydrogen bonds with either of them in CDK2 were completely identical. The same happened to the arginine residue at the 424th location of MMP9 when docking with quercetin or diosmetin.

## Discussion

Bladder cancer is the most common malignancy of the urinary system with painless gross hematuria as the cardinal symptom; however, patients may be asymptomatic in the early stage, leading to delayed diagnosis and eventual progression of the disease (van der Heijden and Witjes, 2009). While early detection depends primarily on imaging technologies, some patients are diagnosed as advanced BCa in the first place (Gakis, 2020). Furthermore, men are generally thought to be at higher risk for BCa than women. This gender pattern of incidence persists worldwide, but divided trends have been observed, particularly in developed countries, with consistent rates in men and increasing tendencies among women (Sung et al., 2021). The rising prevalence of smoking in women may partly explain this. The focus of management for BCa should hence be on the prevention of relapse, progression into the advanced stage, and metastasis, as non-muscle invasive BCa is always associated with a promising prognosis, whereas muscle invasive BCa remains refractory, regardless of whether the bladder has been resected or not (van der Heijden and Witjes, 2009). Although great progress has been made in treatment, for instance the emergence of brand-new adjuvant immunotherapy, only a small number of patients benefit from it, not to mention the toxic side effects it brings about (Rey-Cárdenas et al., 2021). With its precious clinical treatment experience and abundant theoretical resources dating back to ancient China, TCM could be a perfect complementary remedy and a key entry point for the development of natural medicines with multi-target potential.

From the perspective of TCM, the bladder is an organ responsible for eliminating the “damp evil” that arises daily in the human body. Should the bladder fail to eliminate it, it would accumulate and transform into a combination of damp and heat evils, eventually causing the cancer together, during which time the two evils could probably block neighboring vessels. This is how hematuria comes into being, according to TCM theory. The botanical medicine in LSYQ Decoction serve precisely the TCM principle of clearing the heat and prompting diuresis. *Solanum Nigrum Linn.*, a herbal plant rich in flavonoids, is widely adopted in TCM for its antineoplastic effect (Butt et al., 2018). Previous studies shown that alpha-solanine, an extract of *Solanum Nigrum Linn.*, could inhibit invasion of human prostate cancer cells by suppressing epithelial-mesenchymal transition and matrix metalloproteinase (MMP) expression (Shen et al., 2014), while *Solanum Nigrum Linn.* Polysaccharide-1, another active ingredient therein, exhibited immunomodulatory activity by promoting the release of NO, tumor necrosis factor-α and interleukin (IL)-6 in macrophages and inducing Th1 responses mediated by IL -2 and interferon-γ (Pu et al., 2020). Extracts of *Duchesnea Indica Focke*, also known as mock strawberry, have been reported to have anti-metastatic potential by prohibiting MMP2 activity in human oral squamous cells (Yang et al., 2019). As one of the most important elements in Baiying Qinghou Decoction, *Herba Solani Lyrati* (Baiying in Chinese) could target *TP53, EGFR, IL1B and NOS3* proteins for the treatment of laryngeal squamous cell carcinoma (Gao et al., 2021). *Smilacis Glabrae Rhizoma*, a member of the Smilacaceae family native to Southeast Asia, has long been utilized to treat sore throat and syphilis. It also exhibited an anti-tumor effect in a dose-dependent manner in human breast cancer cell line MCF7, colon carcinoma cell line HT -29, and gastric cancer cell line BGC-823 by inducing mitochondrial apoptosis (Gao et al., 2011). *Juncus effusus L.* has been attributed with a variety of pharmacological functions, including anti-inflammatory, anti-allergic, anti-cancer and others (Ma et al., 2016; Park et al., 2016). For example, dehydroeffusol, a low molecular weight phenanthrene from *Juncus effusus L.*, effectively induced endoplasmic reticulum stress, resulting in decreased proliferation of gastric cancer cells (Zhang et al., 2016). Nevertheless, the effect of LSYQ Decoction on bladder cancer has not been clearly elucidated.

Considering the composition diversity of this formula, we made use of network pharmacology to systemically explore it at herbal, molecular, and genetic levels in the present study. Upon strict screening, 38 active compounds were finally identified, among which 654 human proteins were predicted as potential targets. It again proved that TCM formulas possess the characteristics of multi-drug and multi-target. The TCM targets are intersected with BCa-related protein-coding genes *via* a Venn diagram. The hub target genes, namely CDK2, EGFR, MMP9 and PTGS2, were then determined through the visualization of the PPI network and module analysis. Meanwhile, quercetin, diosmetin, enhydrin, as well as luteolin were regarded as core constituents in LSYQ Decoction through topological mining. The target genes in both of the two modules, whose cluster scores were greater than 10, have accumulated in significant pathways related to cancers, such as the PI3K-Akt signaling pathway, the ErbB signaling pathway, the VEGF signaling pathway, the MAPK signaling pathway, and the HIF-1 signaling pathway.

The anti-tumor activity of flavonoids has been extensively described and widely accepted (Teillet et al., 2008). Quercetin, a flavonoid found in abundance in herbal plants and daily consumed foods, can not only restrain the proliferation and induce the apoptosis of various tumor cells via different pathways (Su et al., 2016; Mirazimi et al., 2022; Reyes-Avendaño et al., 2022), but can also act as a free-radical scavenger to exert antioxidant and anti-inflammatory effects, therefore, inhibiting oncogenesis (Hsieh et al., 2022)and inflammatory diseases (Yang et al., 2022). Citrus fruits and extracts of many herbs are also rich in diosmetin (Roowi and Crozier, 2011), another commonly encountered flavone with numerous pharmacological functions, such as anti-inflammation, anti-oxidative stress, and anti-neoplasia (Roma et al., 2018; Chen et al., 2020; Ma and Zhang, 2020). Luteolin, also a natural flavonoid, has been proven to play an important role in anti-inflammatory, antioxidant, antiallergic, and anticancer processes (Juszczak et al., 2022). It could suppress the growth of human bladder cancer cell line T24 by upregulating p21 and inhibiting mTOR signaling (Iida et al., 2020). According to the two PPI networks, the degrees of CDK2, EGFR, *MMP9* and PTGS2 were among the highest, suggesting their roles as hub targets. EGFR overexpression is found in various human malignancies (Levantini et al., 2022), including BCa (Seidl, 2020), and a few monoclonal antibody drugs have been invented to target its protein in breast cancer and non-small cell lung cancer, either approved by the Food and Drug Administration of the US or under clinical trials. CDK2 is a member of the cyclin-dependent kinase family that combines with cyclin E and A in turn during the cell cycle to prompt the transition from the G1 phase to the S phase (Hume et al., 2020), making it a controller that regulates mitosis and cell proliferation. MMP9, a protein from the calcium-dependent zinc-containing endopeptidase family, is engaged in the degradation of extracellular matrix, especially type-IV collagen, resulting in multiple physiological and pathological processes, including tissue remodeling, angiogenesis, cell migration, rheumatoid arthritis, and tumor invasion (Dufour et al., 2010; Xue et al., 2014; Li et al., 2021). Higher MMP9 expression is associated with a poorer prognosis for patients with breast cancer or colorectal cancer (Dufour et al., 2011; Pezeshkian et al., 2021). PTGS2, also known as cyclooxygenase-2, converts arachidonic acid to prostaglandin endoperoxide H2 and engages in downstream inflammatory responses. Not only is the expression of PTGS2 remarkably upregulated during inflammation, but also in various tumors such as gallbladder, and breast cancers (Legan, 2010; Glover et al., 2011). What’s more, prostaglandin E2, the product of PTGS2, was reported to stimulate tumor progression (Menter et al., 2010). In addition to the four targets mentioned above, there are many other proteins participating in the occurrence and progression of BCa that also scored high in Degree values in the two PPI networks, such as AKT1, SRC, CDK1, ESR1, CCNB1, and so on. Ultimately, molecular docking was employed to examine the binding affinity of typical core compounds and hub targets. With the exception of enhydrin, the results suggested that all of the typical compounds had a relatively good binding capacity to four target proteins (affinity less than -5 kcal/mol), and the four molecules even shared stronger bonds with CDK2 and MMP9 with all the binding energies less than -7 kcal/mol (Alam MS. et al., 2022). Molecular docking analysis has increasingly become an important *in-silico* tool to preliminarily check the structural fitness between target proteins and small molecules regardless whether the latter were newly synthesized or repurposed for a different disease (Alam M. S. et al., 2022; Majid et al., 2022).

Our study still leaves some room for improvement. First, both the components of LSYQ Decoction and BCa-related genes were retrieved from databases. We tried our best to search for all of them and set a rational threshold for filtration. Second, the targets of LSYQ Decoction were predicted by a reverse-docking algorithm at SwissPredictionTarget. We cross-validated them by molecular docking. Last but not least, the mechanisms discovered by systemic network pharmacology are still not complete and need further experimental exploration and verification.

## Data Availability

The original contributions presented in the study are included in the article/Supplementary Material, further inquiries can be directed to the corresponding author.

## Abbreviations

BCa, Bladder cancer; BP Biological Process; CC, Cellular Component; DL, Drug-likeness; GO, Gene Ontology; HIF, Hypoxia-inducible factor; IL, Interleukin; KEGG, Kyoto Encyclopedia Genes and Genomes; LSYQ, Longsheyangquan; MF, Molecular Function; mTOR, Mechanistic target of rapamycin kinase; OB, Oral bioavailability; PPI, Protein-protein interaction; TCM, Traditional Chinese Medicine; *VEGF*, Vascular endothelial growth factor; *CDK1*, Cyclin-dependent kinase one; *CDK2*, Cyclin-dependent kinase two; *EGFR*, Epidermal growth factor receptor; *MMP9*, Matrix metalloproteinase nine; *PTGS2*, Prostaglandin-endoperoxide synthase two; *ESR*, Estrogen receptor one.; *CCNB1*, Cyclin B1.
